# High-Entropy Thermistor Ceramics (La_1/3_Nd_1/3_*M*_1/3_)_2_(Zr_1/2_Sn_1/2_)_2_O_7_ (*M* = Sm, Eu, Gd, or Dy) with High Sensitivity for High-Temperature Measurements

**DOI:** 10.3390/s24237523

**Published:** 2024-11-25

**Authors:** Yian Chen, Tingting Xuan, Xiaohui Li, Yuling Tuo, Xiaoyi Chen, Bo Gao

**Affiliations:** 1 Xinjiang Key Laboratory of Electronic Information Materials and Devices, Xinjiang Technical Institute of Physics and Chemistry of CAS, Urumqi 830011, China; cacsz1999@126.com (Y.C.); xuantingting20@mails.ucas.ac.cn (T.X.); lixiaohui@ms.xjb.ac.cn (X.L.); tuoyl@ms.xjb.ac.cn (Y.T.); 2 Center of Materials Science and Optoelectronics Engineering, University of Chinese Academy of Sciences, Beijing 100049, China

**Keywords:** pyrochlore-type structure, negative temperature coefficient (NTC), high entropy, high sensitivity, aging properties

## Abstract

A series of high-entropy pyrochlore ceramics, specifically (La_1/3_Nd_1/3_*M*_1/3_)_2_(Zn_1/2_Sn_1/2_)_2_O_7_ (*M* = Sm, Eu, Gd, or Dy), have been synthesized using the solid-state reaction method. Their potential as high-temperature thermistors was investigated by analyzing electrical and aging properties at elevated temperatures. Characterization using X-ray diffraction, scanning electron microscopy, and Raman spectroscopy confirms that these ceramics are dense, single-phase solid solutions with a pyrochlore structure. Electrical analysis demonstrate that these ceramics maintain high resistivity and resistance stability, exhibiting typical negative temperature coefficient features and high B values across a wide temperature range. These characteristics make (La_1/3_Nd_1/3_*M*_1/3_)_2_(Zn_1/2_Sn_1/2_)_2_O_7_ promising candidates for the development of high-sensitivity, long-life high-temperature thermistors suitable for applications within the temperature range of 400–1200 °C.

## 1. Introduction

Temperature monitoring and control are essential in both daily life and industrial production, with temperature sensors being the key elements that ensure accurate monitoring and regulation of temperature. Among various types of temperature sensors, negative temperature coefficient (NTC) thermistors stand out due to their high sensitivity, simple structure, low cost, and ease of mass production, which leads to their extensive application in industries such as manufacturing, household appliances, and healthcare [1]. With the rapid advancement of sectors like automotive, aerospace, and metallurgy, the demand for NTC thermistors that can operate stably in high-temperature environments is steadily increasing. These thermistors, with their excellent cost-effectiveness, are regarded as ideal substitutes for high-temperature thermocouples composed of precious metals such as platinum and rhodium. However, this is no easy task, as it requires these thermistors to maintain high sensitivity across a broad operating temperature range and to operate reliably over the long term, even at temperatures as high as 1200 °C [2,3].

The thermistor properties are derived from thermistor ceramics. For thermistors to operate stably over extended periods at higher temperature limits and maintain high sensitivity over a wide temperature range, it is essential that the thermistor ceramics exhibit high resistivity and stable electrical performance at elevated temperatures, as well as possess a high B value within the operating temperature range [4]. The variety of thermistor ceramics applicable in high-temperature environments is extensive, and among them, perovskite chromates are notable for their adjustable electrical property across a wide temperature range [5]. This property allows them to achieve customized electrical properties and temperature coefficients of resistance by finely controlling their chemical composition or subtly adjusting their crystalline structure to meet various specific high-temperature requirements. However, despite the implementation of these carefully designed strategies, high-temperature thermistor ceramics based on perovskite chromates have only managed to raise their maximum operating temperature to around 1000 °C, while also facing the dual challenges of unsatisfactory sintering density and significant resistance drift in high-temperature environments [6]. This situation undoubtedly poses significant obstacles to practical applications. Therefore, in light of the inherent limitations of perovskite chromates, the exploration of innovative thermistor ceramics has become a critical research topic.

Pyrochlore-structured ceramics (such as zirconates and stannates) have recently garnered considerable attention in the field of high-temperature thermistors. As a novel class of high-temperature thermistor ceramics, they exhibit excellent structural stability and good sintering density at high temperatures and demonstrate a strong linear correlation between electrical properties and temperature over a wide temperature range. Additionally, their electrical properties can be continuously and precisely adjusted through flexible substitution or doping strategies [7,8]. Notably, in comparison to perovskite chromate thermistor ceramics, they exhibit higher resistivity at identical high temperatures, potentially allowing for an extension of their maximum operating temperature beyond 1000 °C. However, these ceramics still face a challenge in high-temperature applications. Despite previous research employing in situ high-temperature XRD technology to investigate the structural stability of pyrochlore zirconates, which confirms that their phase structure can remain stable and undergo no transformation under high-temperature conditions, it is noteworthy that the degree of disorder in the anion sublattice slight increases [9]. This anion sublattice disorder often results in resistance drift at elevated temperatures, which in turn affects their aging characteristics [10]. Therefore, addressing how to improve the stability of the crystal structure and reduce or inhibit resistance drift at high temperatures has become a critical issue. In this paper, we attempt to address this issue by employing a high-entropy strategy. Given that the introduction of multiple principal elements and severe lattice distortions endows high-entropy ceramics with extremely high configurational entropy, this inevitably reduces the Gibbs free energy of lattice disordering in ceramics [11]. Ultimately, by forming an “entropy-stabilized” structure, the disordering of the anion sublattice is effectively suppressed, ensuring that the thermistor ceramics exhibit stable electrical properties at high temperatures. Based on the above, we synthesized a series of pyrochlore-structured ceramics, (La_1/3_Nd_1/3_*M*_1/3_)_2_(Zn_1/2_Sn_1/2_)_2_O_7_ (*M* = Sm, Eu, Gd, or Dy), with high-entropy characteristics. Our experimental results indicate that these high-entropy ceramics exhibit significant improvements in both the upper applicable temperature limit and aging characteristics, while maintaining high sensitivity within a wide temperature range. This positions them as promising candidate materials for the design of thermistors intended to operate within a temperature range of 400–1200 °C.

## 2. Materials and Methods

Analytically pure La_2_O_3_, Nd_2_O_3_, Sm_2_O_3_, Eu_2_O_3_, Gd_2_O_3_, Dy_2_O_3_, ZrO_2_, and SnO_2_ powders were utilized as precursors to synthesize (La_1/3_Nd_1/3_*M*_1/3_)_2_(Zn_1/2_Sn_1/2_)_2_O_7_ (*M* = Sm, Eu, Gd, or Dy) ceramics using a solid-state reaction route and named LNSZSO, LNEZSO, LNGZSO, and LNDZSO, respectively. The detailed procedure involved the following steps. Initially, the precursors were precisely weighed according to the strict stoichiometric ratios and then underwent an 8 h ball-milling process, during which ethanol acted as a dispersant. Afterward, the homogenized mixture underwent drying at 150 °C for 6 h to eliminate any traces of residual solvent, followed by a pre-sintered step at 1200 °C for 4 h to facilitate dense sintering. Subsequently, the pre-sintered powder underwent a re-milling process to obtain a refine one. This refined powder was then pressed into cylindrical pellets using a uniaxial oil pressure of 10 MPa and a cold isostatic pressure of 300 MPa in that order, each precisely crafted to a diameter of 10.0 mm and a thickness of 1.5 mm. Finally, these cylindrical pellets were placed in a high-temperature furnace for a 10 h pressureless sintering process at 1600 °C to obtain (La_1/3_Nd_1/3_*M*_1/3_)_2_(Zn_1/2_Sn_1/2_)_2_O_7_ ceramics.

The densities of the samples were determined using Archimedes’ principle, with deionized water serving as the immersion medium. To ascertain the crystal structure of the samples, X-ray diffraction (XRD) analysis was executed on a Bruker D8 Advance A25 diffractometer (Germany) using Cu Kα radiation (λ = 1.5406 Å). The XRD patterns were collected over a 2θ range from 10° to 100°, with a step size of 0.02° and a dwell time of 0.2 s per step. The acquired XRD patterns were subsequently analyzed utilizing the Rietveld refinement method within the General Structure Analysis System (GSAS-II) software. Raman spectral analysis was conducted with a Horiba LabRAM HR Evolution (France) spectrometer. To observe the surface morphology and elemental distribution of the samples, scanning electron microscopy (SEM) observations and energy dispersive spectroscopy (EDS) analysis were conducted on a Zeiss Supra 55 VP (Germany) microscope equipped with the Quantax 200 system. The chemical valence states of stannum were analyzed using X-ray photoelectron spectroscopy (XPS) with Al Kα radiation performed on a Thermo Scientific (America) Kα+ instrument. For electrical characterization, a uniform layer of conductive platinum paste was applied to both faces of the sintered pellets, followed by an annealing process at 1200 °C to fabricate the electrodes. An Agilent 34970A digital multimeter (America) was utilized to measure the resistance of the electrodes within a temperature range of 400–1200 °C, generating the resistance–temperature curve of the corresponding ceramics. To evaluate the electrical stability of the samples, an aging test was performed by exposing the electrodes to a tube furnace at 1200 °C for an extended period of 500 h. The relative resistance drift (ΔR/R_0_) was calculated using the following formula:

ΔR/R_0_ = (R − R_0_)/R_0_ (1)

where R and R_0_ represent the resistance value of the electrodes before and after aging.

The special quasi-random structure (SQS) modeling method was firstly employed to construct a 3 × 1 × 1 pyrochlore supercell structure with 66 atoms and containing three rare earth elements, including La, Nd, and *M* (*M* = Sm, Eu, Gd or Dy) [12]. In this study, the 4f electrons of all rare earth elements were considered as part of the core electrons. Subsequently, the Vienna ab initio simulation package (VASP 6.3.0) was used to perform first-principle calculations based on density functional theory (DFT) [13]. The generalized gradient approximation (GGA) parameterized using the Perdew–Burke–Enrzerhof (PBE) was used to describe the exchange-correlation effect [14]. The ion–electron interactions were treated using the projector augmented-wave (PAW) method [15]. The calculations were performed with a 2 × 2 × 2 k-point sampling in the reciprocal space [16]. The plane wave basis cutoff energy and the convergence criterion for the total energy were 520 eV and 10^−5^ eV, respectively.

## 3. Results and Discussion

**Figure 1:**
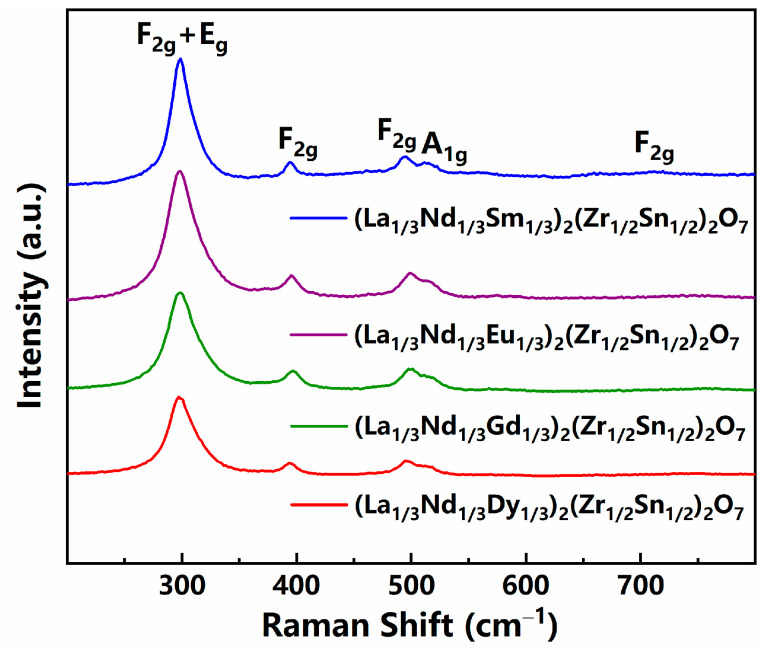
Raman spectra of the (La_1/3_Nd_1/3_*M*_1/3_)_2_(Zn_1/2_Sn_1/2_)_2_O_7_ ceramics.

For A_2_B_2_O_7_ oxides, empirical observations have shown that a pyrochlore structure often forms when the cationic radius ratio (r_A_/r_B_) of the A and B sites falls within the range of 1.46 to 1.78 [17]. Below this range, a defective fluorite structure is typically formed. Since the r_A_/r_B_ values in the (La_1/3_Nd_1/3_*M*_1/3_)_2_(Zn_1/2_Sn_1/2_)_2_O_7_ (*M* = Sm, Eu, Gd, or Dy) ceramics, calculated using the average radii of the A and B site cations, fall between 1.558 and 1.583, it is expected that these materials will exhibit characteristics of a pyrochlore structure. To verify this, we conducted Raman spectroscopy tests on the (La_1/3_Nd_1/3_*M*_1/3_)_2_(Zn_1/2_Sn_1/2_)_2_O_7_ ceramics, and the results are shown in Figure 1. Typical pyrochlore structure ceramics exhibit six distinct Raman active modes in the 200–800 cm^−1^ wavelength range, whereas defective fluorite structure ceramics produce broadened and weaker vibrational modes due to increased disorder [18]. As shown in Figure 1, six distinct active modes are clearly observed in the Raman spectra of all ceramics. Specifically, the peak located around 298 cm^−1^, representing the E_g_ mode, can be classified as an O-A-O bending vibration. The peaks situated near 395 cm^−1^ and 517 cm^−1^, which signify the F_2g_ mode, can be categorized as B-O stretching vibrations. Furthermore, the peak positioned around 499 cm^−1^, indicative of the A_1g_ mode, can be identified as an A-O stretching vibration. These findings confirm the pyrochlore structural characteristics of these four ceramics.

**Figure 2:**
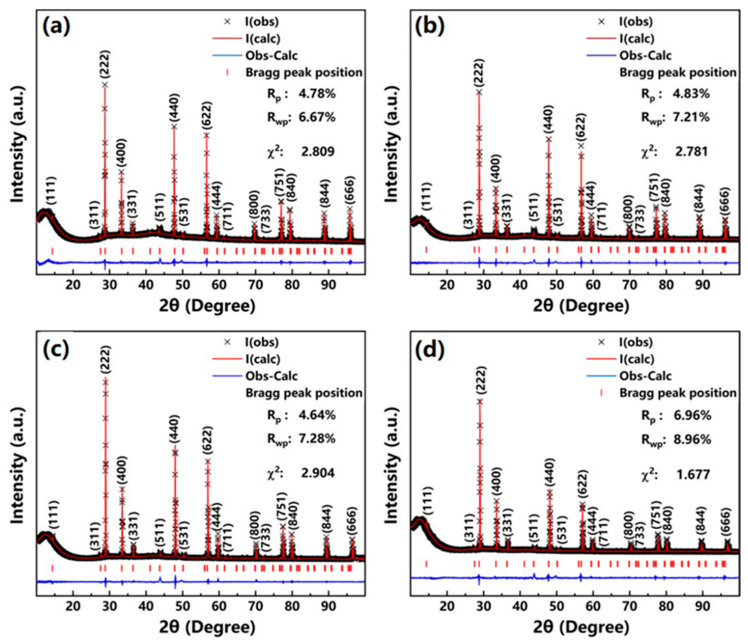
The XRD patterns and Rietveld refinement results for the (La_1/3_Nd_1/3_*M*_1/3_)_2_(Zn_1/2_Sn_1/2_)_2_O_7_ ceramics: (**a**) LNSZSO; (**b**) LNEZSO; (**c**) LNGZSO; (**d**) LNDZSO.

The pyrochlore structure of the (La_1/3_Nd_1/3_*M*_1/3_)_2_(Zn_1/2_Sn_1/2_)_2_O_7_ ceramics can also be confirmed using XRD. Figure 2 presents the XRD patterns of these ceramics along with their Rietveld refinement results. The XRD patterns of the four ceramics exhibit distinct diffraction peaks indicative of a pyrochlore structure at 2θ angles of 29°, 34°, 48°, and 57°. In particular, a series of weak superlattice diffraction peaks can be observed at approximately 14.2°, 27.4°, 36.3°, 43.6°, 49.9°, 61.3°, and 66.5°, which are key criteria for distinguishing the pyrochlore structure from the defective fluorite structure, despite their shared A_2_B_2_O_7_ chemical formula [19]. This observation conclusively verifies that the (La_1/3_Nd_1/3_*M*_1/3_)_2_(Zn_1/2_Sn_1/2_)_2_O_7_ ceramics possess a pyrochlore structure. Additionally, during the sintering process of multi-element co-doped oxide ceramics, the formation of multiple phases with the same structure may occur due to the disordered distribution of cationic [20]. To verify the phase characteristics of these ceramics, we further refined their XRD spectra for single-phase analysis. The experimental data obtained aligned well with the theoretical calculations, with R_p_ and R_wp_ values both below 10%. This supports the conclusion that each ceramic exhibits a single-phase pyrochlore structure, which aligns with the analytical results obtained from Raman spectrum analysis. In this case, we calculate the configurational entropy for the four ceramics using the following formula:

(2) $$\Delta S_{con}=-R\sum_{i}^{N}x_{i}lnx_{i}=-RlnN^{-1}=RlnN$$

where x_i_ denotes the mole fraction of the i-th element and N denotes the total count of different elements at specified lattice sites. The computed configurational entropy for the four ceramics is found to be 1.79R, notably exceeding the 1.5R threshold used as the criterion for classifying materials as high-entropy ceramics [11]. This result, coupled with the Raman and XRD characterization outcomes, confirms that the (La_1/3_Nd_1/3_*M*_1/3_)_2_(Zn_1/2_Sn_1/2_)_2_O_7_ ceramics are high-entropy ceramics featuring a single-phase pyrochlore structure.

**Figure 3:**
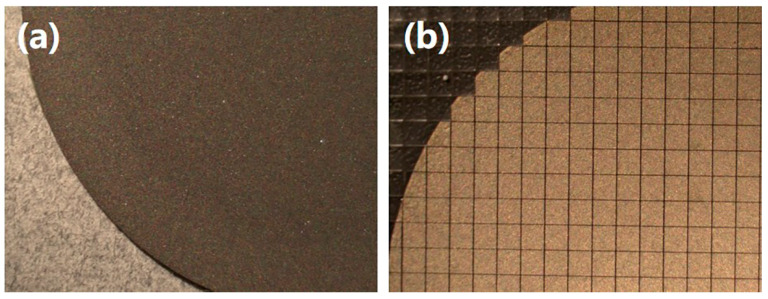
The (**a**) wafers and (**b**) chips of (La_1/3_Nd_1/3_Sm_1/3_)_2_(Zn_1/2_Sn_1/2_)_2_O_7_ ceramic obtained after slicing and dicing.

**Figure 4:**
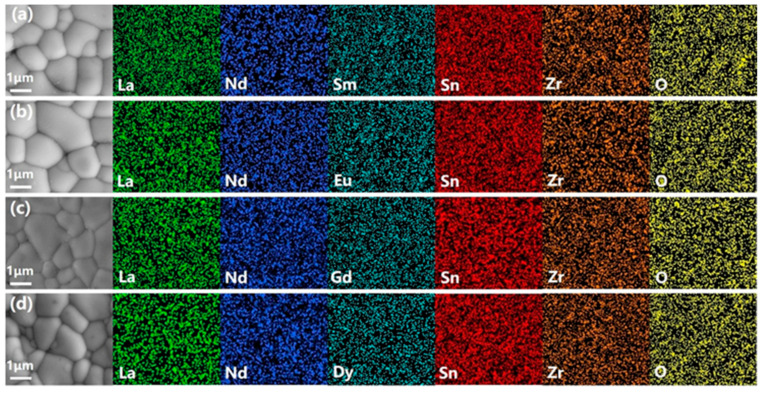
SEM images and EDS mapping of the (La_1/3_Nd_1/3_*M*_1/3_)_2_(Zn_1/2_Sn_1/2_)_2_O_7_ ceramics: (**a**) LNSZSO; (**b**) LNEZSO; (**c**) LNGZSO; (**d**) LNDZSO.

Typically, the mass production of thermistors involves precise processes such as slicing and dicing. During these operations, diamond blades are employed to cut ceramic ingots into thick wafers and subsequently dice these wafers into chips. Therefore, achieving high sintering density and homogeneous element distribution in the ceramics is crucial for ensuring batch consistency in the electrical performance of the thermistors. However, most high-temperature NTC thermistor ceramics present significant challenges during these manufacturing processes due to their inherent brittleness and the presence of non-dense defects [21]. These characteristics can give rise to the formation of cracks and fractures within the wafers and chips, thereby compromising the quality and reliability of the final product [22]. Hence, we select the (La_1/3_Nd_1/3_Sm_1/3_)_2_(Zn_1/2_Sn_1/2_)_2_O_7_ ceramic pellets for slicing and dicing and successfully produced wafers and small square chips. These wafers and chips exhibit high integrity with no obvious cracks, as shown in Figure 3. Additionally, the typical surface micrographs of the sintered (La_1/3_Nd_1/3_*M*_1/3_)_2_(Zn_1/2_Sn_1/2_)_2_O_7_ ceramics are shown in Figure 4. It can be observed that all ceramics exhibit a dense microstructure with clear grain boundaries. The relative density of all ceramics, as measured using the Archimedes method, is greater than 98%. The EDS analysis results for these ceramics, as shown in Figure 4, indicate that each element is uniformly distributed within the respective ceramics. These characteristics not only ensure the consistency of the NTC chips during batch processing but also demonstrate the potential of these NTC ceramics in achieving large-scale production.

**Figure 5:**
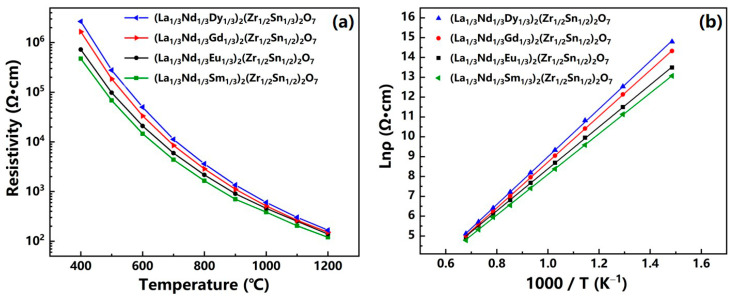
(**a**) Plot of resistivity vs. temperature and (**b**) relationship between lnρ and 1000/T for the (La_1/3_Nd_1/3_*M*_1/3_)_2_(Zn_1/2_Sn_1/2_)_2_O_7_ ceramics.

**Figure 6:**
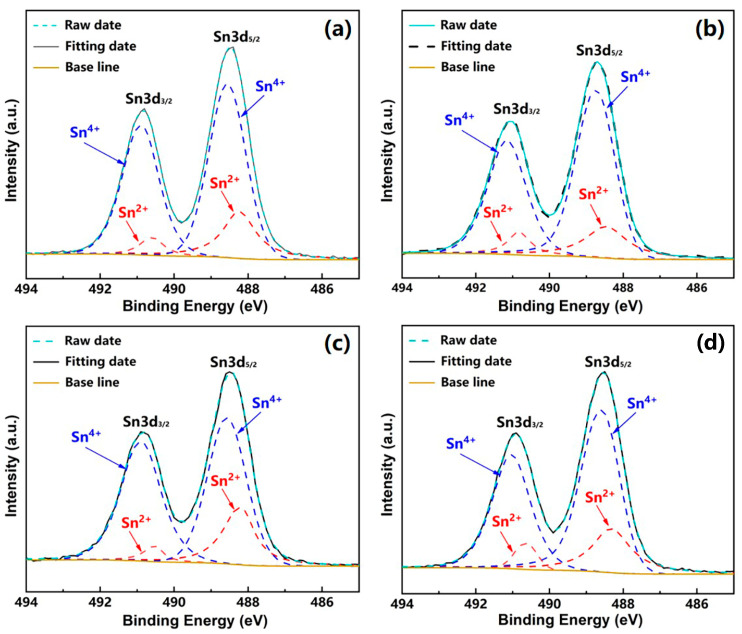
XPS image of the Sn3d orbital of the (La_1/3_Nd_1/3_*M*_1/3_)_2_(Zn_1/2_Sn_1/2_)_2_O_7_ ceramics: (**a**) LNSZSO; (**b**) LNEZSO; (**c**) LNGZSO; (**d**) LNDZSO.

Figure 5a illustrates the temperature dependence of the resistivity for the (La_1/3_Nd_1/3_*M*_1/3_)_2_(Zn_1/2_Sn_1/2_)_2_O_7_ ceramics. The curves demonstrate an exponential decrease in resistivity with increasing temperature, indicative of the NTC characteristic. Figure 6 displays the XPS spectra of Sn 3d signals for these ceramics. Using Gaussian–Lorentzian fitting of the XPS spectra, it is found that the characteristic peaks arise from the superposition of two sets of peaks, corresponding to different oxidation states of stannum ions. Peaks at binding energies of 491 eV and 488.6 eV are attributed to the 3d_3/2_ and 3d_5/2_ states of Sn^4+^, while those at 490.6 eV and 488.3 eV are associated with the 3d_3/2_ and 3d_5/2_ states of Sn^2+^ [23]. The presence of Sn^2+^ ions is attributed to the capture of electrons by Sn^4+^ ions occupying the six coordination from adjacent O^2−^ during the high-temperature sintering process, leading to the formation of Sn^2+^ along with the simultaneous generation of holes. As the temperature increases, the migration of these holes accelerates, leading to a decrease in resistivity, thereby exhibiting NTC characteristics. Additionally, comparisons indicate a gradual decrease in the peak intensity of Sn^2+^ in these ceramics, signaling a reduction in the relative content of Sn^2+^. This decline implies a decrease in hole concentration, leading to a progressive reduction in resistivity from LNSZSO to LNEZSO, LNGZSO, and ultimately to LNDZSO.

For thermistors, temperature measurement involves reading the resistance value, then retrieving the corresponding temperature from a predefined temperature resistance reference table, and finally outputting the corresponding temperature signal [17]. Since this table is typically established based on the mathematical relationship between the resistance values of the thermistor at several reference temperatures, the closer the measured resistance value aligns with this mathematically relationship, the higher the measurement accuracy of the thermistor will be. As depicted in Figure 5b, the natural logarithm of resistivity (lnρ) for these four ceramics demonstrates a linear correlation with the inverse of temperature (1000/T), characterized by linear fitting coefficients exceeding 0.999. This suggests that the difference between the apparent temperature obtained from the temperature resistance table based on the linear lnρ-1000/T relationship and the actual temperature is nearly negligible. This ensures a high measurement accuracy for thermistors fabricated from this series of ceramics.

As previously mentioned, for thermistors to be applicable at higher temperatures, they need to maintain high resistivity at elevated temperatures [24]. For perovskite chromate ceramics, their resistivity undergoes a sharp decline to merely a few tens of Ω·cm near 1000 °C, making them inadequate for use in environments requiring higher temperatures [2,3,25]. However, as shown in Figure 5a, the resistivity of the (La_1/3_Nd_1/3_*M*_1/3_)_2_(Zn_1/2_Sn_1/2_)_2_O_7_ ceramics at 1000 °C is significantly higher than that of perovskite high-temperature thermistor ceramics, which is only a few tens of Ω·cm at the same temperature, almost an order of magnitude higher. This trend persists even at high temperatures of 1200 °C, with values of 166.8 Ω·cm, 151.4 Ω·cm, 139.5 Ω·cm, and 119.6 Ω·cm, respectively, nearly triple that of perovskite ceramics. Thus, the high resistivity at high temperatures exhibited by the (La_1/3_Nd_1/3_*M*_1/3_)_2_(Zn_1/2_Sn_1/2_)_2_O_7_ ceramics, combined with the previously noted highly linear relationship between lnρ and 1000/T, makes them suitable for application in the temperature range from 400 to 1200 °C. Additionally, the material constant B value can be calculated based on the slope of lnρ versus 1000/T. Calculations demonstrated that the B value for the (La_1/3_Nd_1/3_*M*_1/3_)_2_(Zn_1/2_Sn_1/2_)_2_O_7_ ceramics are 10,233 K, 10,554 K, 11,604 K, and 12,071 K, respectively. We note that these values are approximately twice as high as those of perovskite high-temperature thermistor ceramics, which range around 4000 K [2,3,25]. Considering that the sensitivity of thermistors is positively correlated with their B value, this finding suggests that the (La_1/3_Nd_1/3_*M*_1/3_)_2_(Zn_1/2_Sn_1/2_)_2_O_7_ ceramics exhibit exceptional sensitivity within their operating temperature range.

**Figure 7:**
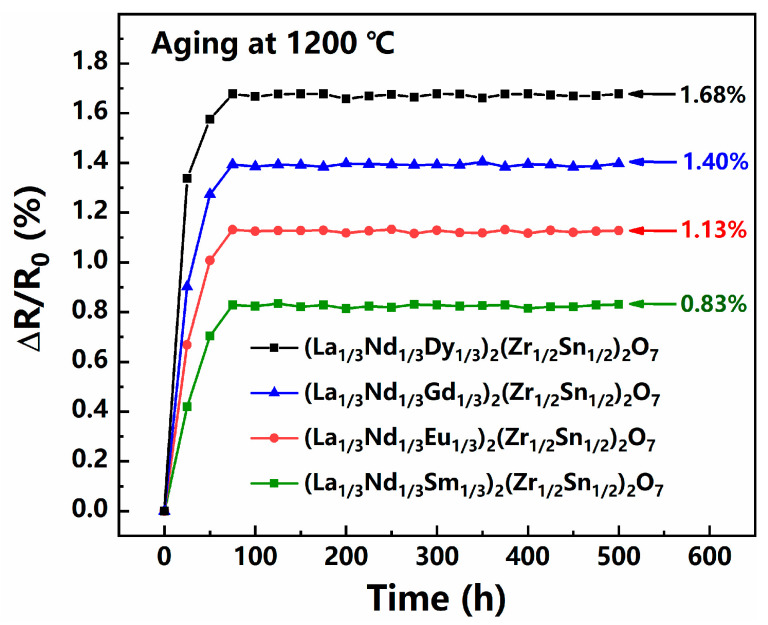
Plot of the resistance drift (ΔR/R_0_) and aging time for the (La_1/3_Nd_1/3_*M*_1/3_)_2_(Zn_1/2_Sn_1/2_)_2_O_7_ ceramics.

**Figure 8:**
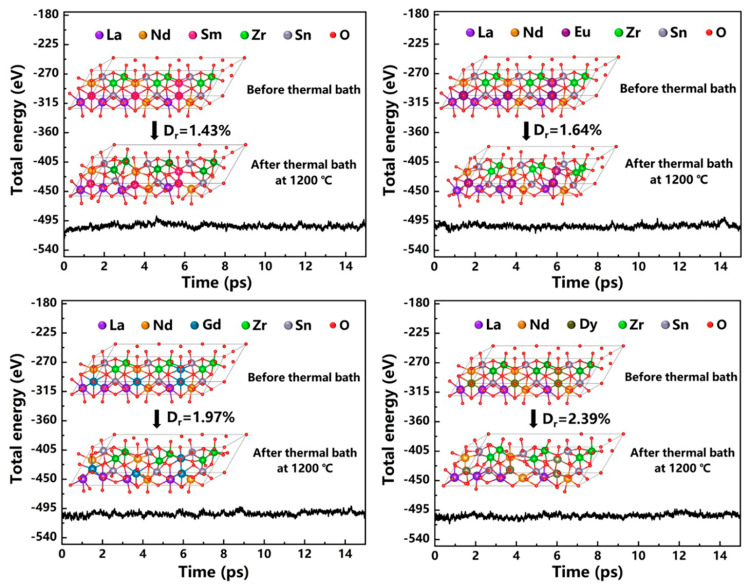
The total energy fluctuations of the (La_1/3_Nd_1/3_*M*_1/3_)_2_(Zn_1/2_Sn_1/2_)_2_O_7_ ceramics at 1200 °C (the insets present snapshots of the crystal structures before and after thermal bath).

If NTC thermistors are to operate at higher temperatures, thermistor ceramics are not only required to maintain high resistivity at high temperatures but also to ensure the sustained stability of electrical performance. As shown in Figure 7, the resistance of the (La_1/3_Nd_1/3_*M*_1/3_)_2_(Zn_1/2_Sn_1/2_)_2_O_7_ ceramics drifted by only 0.83%, 1.13%, 1.40%, and 1.68%, respectively, after being exposed to 1200 °C for 500 h. Compared to the significant 20% to 50% resistance drift experienced by perovskite chromite-based high-temperature thermistor ceramics at their maximum applicable temperature limit (1000 °C), the drift exhibited by the four thermistor ceramics has been notably improved [2,3,25]. This comparison highlights the outstanding aging performance of the (La_1/3_Nd_1/3_*M*_1/3_)_2_(Zn_1/2_Sn_1/2_)_2_O_7_ ceramics, demonstrating their higher electrical stability in high-temperature environments. It is worth noting that, as shown in Figure 7, resistance drift primarily occurs within the first 75 h and stabilizes thereafter. Since anion sublattice disorder is the primary contributor to resistance drift at high temperatures in pyrochlore-structured thermistor ceramics, we first constructed 3 × 1 × 1 supercells for the four ceramics and subsequently performed ab initio molecular dynamics (AIMD) simulations (canonical ensemble and Andersen thermostat) at 1200 °C. We monitored the total energy fluctuations and structural variations of these supercells over a 15-picosecond period [26]. As shown in Figure 8 and its inset, slight deviations in the atomic positions within the supercell can be observed after the thermal bath. This is attributed to lattice distortion caused by the increase of atomic relaxation movements at high temperatures. We calculated the relative distortion (*D_r_*) that represents the degree of atomic deviation from their equilibrium lattice positions in the crystal structure according to the formula below [27]:

(3) $$D_{r}=\frac{\sum_{i=1}^{n}\sqrt{{\left(x_{i,II}-x_{i,I}\right)}^{2}+{\left(y_{i,II}-y_{i,I}\right)}^{2}+{\left(z_{i,II}-z_{i,I}\right)}^{2}}}{\left(n-1\right)\times d_{0}}\times 100\%$$

where $x_{i,I}$ and $x_{i,II}$ represent the position of the atom before and after the thermal bath, respectively; *d*_0_ represents the distance of the nearest atom pair in the optimized structure; and *n* is the total number of atoms in the calculated supercell. In this study, *n* is 66. Since we fixed an atomic coordinate as the origin in 66 atoms, the number of twisted atoms involved is *n* − 1. The calculated *D_r_* for the four ceramics is 1.43%, 1.64%, 1.97%, and 2.39%, respectively. Clearly, these distortions in the lattice structure will affect the transport of charge carriers, thereby causing resistance drift in the ceramics. However, the total energy of the supercells for the four ceramics remained stable, indicating that they still possess good structural stability even at 1200 °C. This stability arises from the high configurational entropy of high-entropy ceramics, which reduces the Gibbs free energy associated with lattice distortions, effectively preventing further structural deformation at high temperatures and allowing the lattice structure to reach a new thermodynamic equilibrium state under sustained high temperatures [28]. As a result, the resistance values remain stable over longer periods thereafter.

## 4. Conclusions

In this study, a series of high-temperature thermistor ceramics (La_1/3_Nd_1/3_*M*_1/3_)_2_(Zn_1/2_Sn_1/2_)_2_O_7_ (*M* = Sm, Eu, Gd, or Dy) featuring a pyrochlore structure were synthesized using a solid-phase method. Our research has found that these ceramics exhibit high resistivity and excellent electrical stability under high-temperature conditions, allowing them to operate reliably in extreme high-temperature environments up to 1200 °C over extended periods. Furthermore, they also have a high material constant B value and exhibit a highly linear correlation between the logarithm of resistivity and the inverse of temperature over a wide temperature range. This ensures that these ceramics maintain high sensitivity performance within their working temperature range of 400 °C to 1200 °C. In light of these characteristics, (La_1/3_Nd_1/3_*M*_1/3_)_2_(Zn_1/2_Sn_1/2_)_2_O_7_ ceramics provide a promising material solution for developing high-performance, low-cost high-temperature thermistors.

## Data Availability

Data are contained within the article.
